# Investigational treatment of rheumatoid arthritis with a vibrotactile device applied to the external ear

**DOI:** 10.1186/s42234-019-0020-4

**Published:** 2019-04-17

**Authors:** Meghan E. Addorisio, Gavin H. Imperato, Alex F. de Vos, Steve Forti, Richard S. Goldstein, Valentin A. Pavlov, Tom van der Poll, Huan Yang, Betty Diamond, Kevin J. Tracey, Sangeeta S. Chavan

**Affiliations:** 1 Center for Biomedical Science and Bioelectronic Medicine, Feinstein Institute for Medical Research, Northwell Health, Manhasset, NY USA; 2 Elmezzi Graduate School of Molecular Medicine, Feinstein Institute for Medical Research, Northwell Health, Manhasset, NY USA; 3 Donald and Barbara Zucker School of Medicine at Hofstra/Northwell, Hempstead, NY USA; 4 Center for Experimental and Molecular Medicine, Academic Medical Center, University of Amsterdam, Amsterdam, The Netherlands; 5 Mass Bay Engineering, Norwell, MA USA; 6 ProHealth Care Associates, Jericho, NY USA; 7 Center for Autoimmune, Musculoskeletal, and Hematopoietic Diseases, Feinstein Institute for Medical Research, Northwell Health, Manhasset, NY USA

**Keywords:** Auricular vagus nerve, taVNS, rheumatoid arthritis, TNF

## Abstract

**Background:**

Rheumatoid arthritis (RA) is a chronic and debilitating inflammatory disease characterized by extensive joint tissue inflammation. Implantable bioelectronic devices targeting the inflammatory reflex reduce TNF production and inflammation in preclinical models of inflammatory disease, and in patients with RA and Crohn’s disease. Here, we assessed the effect of applying a vibrotactile device to the cymba concha of the external ear on inflammatory responses in healthy subjects, as well as its effect on disease activity in RA patients.

**Methods:**

Six healthy subjects received vibrotactile treatment at the cymba concha, and TNF production was analyzed at different time points post-stimulation. In a separate study, nineteen healthy subjects were enrolled in a randomized cross-over study, and effects of vibrotactile treatment at either the cymba concha or gastrocnemius on cytokine levels were assessed. In addition, the clinical efficacy of vibrotactile treatment on disease activity in RA was assessed in nine patients with RA in a prospective interventional study.

**Results:**

Vibrotactile treatment at the cymba concha reduced TNF levels, and the suppressive effect persisted up to 24 h. In the cross-over study with 19 healthy subjects, vibrotactile treatment at the cymba concha but not at the gastrocnemius significantly reduced TNF, IL-1β, and IL-6 levels compared to pre-treatment baseline (TNF *p* < 0.05, IL-6 *p* < 0.01, IL-1β *p* < 0.001). In healthy subjects, vibrotactile treatment at the cymba concha inhibited TNF by 80%, IL-6 by 73%, and IL-1β by 50% as compared to pre-treatment baseline levels. In RA patients, a significant decrease in DAS28-CRP scores was observed two days post-vibrotactile stimulation at the cymba concha (DAS28-CRP score pre-treatment = 4.19 ± 0.33 vs post-treatment = 3.12 ± 0.25, *p* < 0.05). Disease activity remained significantly reduced 7 days following vibrotactile treatment (DAS28-CRP score 7 days post-treatment = 2.79 ± 0.21, *p* < 0.01). In addition, a persistent improvement in visual analogue scale scores, a patient derived measure of global health assessment, was observed in RA patients following vibrotactile treatment.

**Conclusion:**

Application of a vibrotactile device to the cymba concha inhibits peripheral blood production of TNF, IL-1β, and IL-6 in healthy subjects, and attenuates systemic inflammatory responses in RA patients.

**Trial registrations:**

ClinicalTrials.gov Identifier: NCT01569789 and NCT00859859. The AMC trial conducted in The Netherlands does not have a ClinicalTrials.gov Identifier.

## Introduction

Rheumatoid arthritis (RA) is a progressive and debilitating disease characterized by an inflammatory pannus whose growth is stimulated by TNF and IL-6 (Smolen et al. 2016). Early work identified anti-TNF monoclonal antibodies as protective against lethality in animal models of septic shock (Tracey et al. 1987), and set the stage for the clinical translation of anti-TNF agents to a variety of inflammatory diseases. Anti-TNF biologics are widely-prescribed and efficacious in rheumatoid arthritis, ankylosing spondylitis, inflammatory bowel disease, and psoriasis. Despite their clinical efficacy, these agents are costly (>$25,000 per patient annually), and render patients susceptible to serious infections as a direct result of TNF blockade (Monaco et al. 2015). To circumvent these limitations of conventional anti-TNF agents, bioelectronic medicine has emerged as a promising alternative approach to target systemic inflammation (Olofsson and Tracey 2017). Bioelectronic devices targeting the inflammatory reflex reduce TNF and inflammation in preclinical models of inflammatory disease, and in patients with rheumatoid arthritis and Crohn’s disease (Chavan et al. 2017; Andersson and Tracey 2012a; Levine et al. 2014; Koopman et al. 2016; Bonaz et al. 2016).

Neural reflexes control the cardiovascular, pulmonary, gastrointestinal, renal, hepatic, and endocrine systems. Recent studies revealed that innate and adaptive immunity are also controlled by neural reflex mechanisms (Chavan et al. 2017; Chavan and Tracey 2017; Pavlov et al. 2018). The vagus nerve-based inflammatory reflex is a physiological mechanism though which the vagus nerve signals regulate immune function (Borovikova et al. 2000; Andersson and Tracey 2012b; Tracey 2002). Molecular mediators of innate immunity activate the afferent signals in the vagus nerve which are transmitted to the brainstem that controls outgoing efferent signals in the vagus nerve. Efferent signals arising in the vagus nerve are then transmitted to the splenic nerve and synapse on splenic lymphocytes causing them to release acetylcholine, which binds the α7 nicotinic acetylcholine receptor (α7nAChR) expressed on macrophages and monocytes (Chavan et al. 2017; Wang et al. 2003; Olofsson et al. 2012). Signal transduction induced by acetylcholine binding increases intracellular calcium, decreases nuclear translocation of NFκB, stabilizes mitochondrial membranes, and inhibits inflammasome activity. These events result in the reduction of the pro-inflammatory cytokines TNF, IL-1β, and IL-6 produced by the spleen (Olofsson et al. 2012). α7nAChR-mediated inhibition of TNF is dependent on CREB and c-FOS (Tarnawski et al. 2018).

Positive pilot clinical trial results have been reported in patients with RA and Crohn’s disease treated with implanted stimulators that deliver electrical impulses to the cervical vagus nerve (Koopman et al. 2016; Bonaz et al. 2016). The vagus nerve is a bilateral cranial nerve which arises from brainstem nuclei and innervates the viscera. The auricular branch of the vagus nerve arises from the vagus and supplies cutaneous regions of the concha and posterior aspect of the lower ear with afferent innervation (Henry 2002; Peuker and Filler 2002; Trevizol et al. 2016; Kong et al. 2018). Transcutaneous auricular vagus nerve stimulation (taVNS) is an investigational therapy in which electrical signals are applied to the cutaneous territory supplied by the auricular branch of the vagus nerve. There are numerous studies of taVNS currently planned or recruiting in the United States and abroad for a range of diseases, including depression, stroke, atrial fibrillation, and heart failure. Functional MRI studies have demonstrated that taVNS activates known brain projections of the vagus, including the nucleus tractus solitarius, dorsal raphe, locus coeruleus, parabrachial area, hypothalamus, amygdala, anterior cingulate cortex, anterior insula, and nucleus accumbens (Yakunina et al. 2017; Kraus et al. 2007; Dietrich et al. 2008; Kraus et al. 2013; Krause et al. 2013; Frangos et al. 2015; Fang et al. 2017). Previous clinical studies have evaluated the effect of taVNS on a variety of conditions and physiological states, including pharmacoresistant epilepsy, depression, pre-diabetes, tinnitus, memory, stroke, and oromotor dysfunction (Kong et al. 2018; Rong et al. 2014; Rong et al. 2016; Stefan et al. 2012; Huang et al. 2014; Shim et al. 2015; Jacobs et al. 2015; Badran et al. 2018; Redgrave et al. 2018). These studies have made use of a range of electrical stimulation settings, as well as several stimulation sites on the auricle or mastoid process, however, it is unknown whether applying a stimulus to the cutaneous region innervated by the auricular branch of the vagus nerve could attenuate systemic inflammatory responses in human subjects. Moreover, it remains unknown whether a mechanical stimulus applied to the cutaneous region innervated by the auricular branch of the vagus nerve, i.e., the cymba concha can attenuate systemic inflammatory responses.

We have reported that transcutaneous mechanical activation of the cervical vagus nerve is therapeutically efficacious in murine models of sepsis (Huston et al. 2007). Pressure applied directly to the skin overlying the cervical vagus nerve dose-dependently attenuated serum TNF levels during lethal endotoxemia. These studies demonstrated that transcutaneous mechanical stimulation of the vagus nerve attenuates serum inflammatory cytokine levels, and enhances survival in model of cytokine-mediated disease. Here we show that application of a vibrotactile device to the cymba concha of the external ear inhibits peripheral blood production of TNF, IL-1β, and IL-6 in healthy subjects, and attenuates disease activity in RA patients.

## Methods

### Vibrotactile device

An oscillatory device (X/Y Axial Stimulator, Mass Bay Engineering, Norwell, MA) was designed and fabricated for the clinical studies. The device consists of a hand-held probe containing a motor which spins an eccentric counterweight, producing radial displacement in a circular pattern at the probe tip. Application of the probe tip to the skin of a human subject is sensed as vibration. The device was powered by a 4602PS DC power supply (MPJA, West Palm Beach, FL). For treatment of study subjects, the device was manually applied by a device operator. It was positioned perpendicular to and with its tip in contact with the cymba concha or gastrocnemius, and applied with a force of 0.15 kg. The specifications of the device were measured using an experimental set-up representative of the device treatment administered to study subjects. All tests were performed at 4VDC; current fluctuated from 0.25A to 0.28A influenced by loading. For testing, the handle of the device was cushioned by a ¼″ thick sleeve of closed cell vinyl foam closely resembling the grip and flexibility of human fingers. Device frequency with zero load was measured to be 6600 RPM on the vertical axis. The frequency and amplitude of the device were recorded by applying the device to tissue-matched durometers (Sorbothane Inc., Kent, OH) overlying a #DS2–110 digital force gauge (Imada Instruments, Northbrook, IL). Durometers were matched to the cymba concha and gastrocnemius by tactile sensation; for the cymba concha, a 30 “A” urethane durometer was used, and for the gastrocnemius, a 30 “00” Sorbothane durometer was used. A microscope with reticle was used to assess amplitude. The frequency of device oscillation was measured using a Strobotac 1531-AB strobe light (General Radio Company, Cambridge, MA). When applied to the 30 “A” urethane cymba concha-matched durometer, device frequency was 10,100 RPM, horizontal amplitude was 0.008″, and vertical amplitude was 0.005″. When applied to the 30 “00” Sorbothane durometer, device frequency was 9330 RPM, horizontal amplitude was 0.015″, and vertical amplitude was 0.005″.

### Study design

**Fig. 1:**
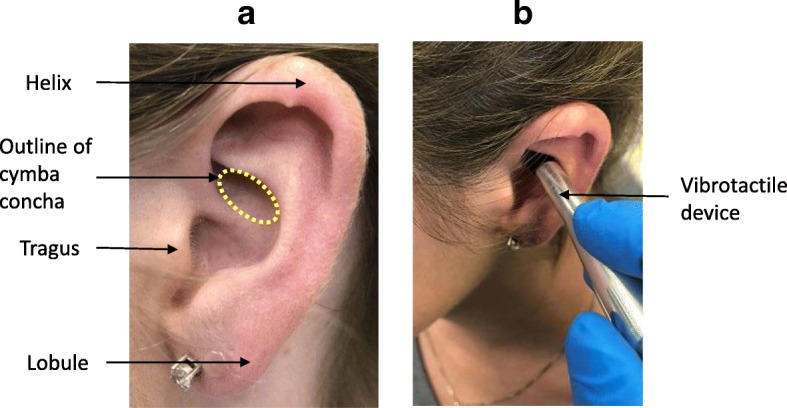
Site of application of vibrotactile device to the cymba concha of the ear. (**a**) Major anatomical landmarks of the external ear (pinna) with approximate outline of the cymba concha. The cymba concha is a highly-conserved anatomical feature of the external ear that was identified by the device operator. (**b**) Representative device placement of the vibrotactile device in contact with the cymba concha

**Fig. 2:**
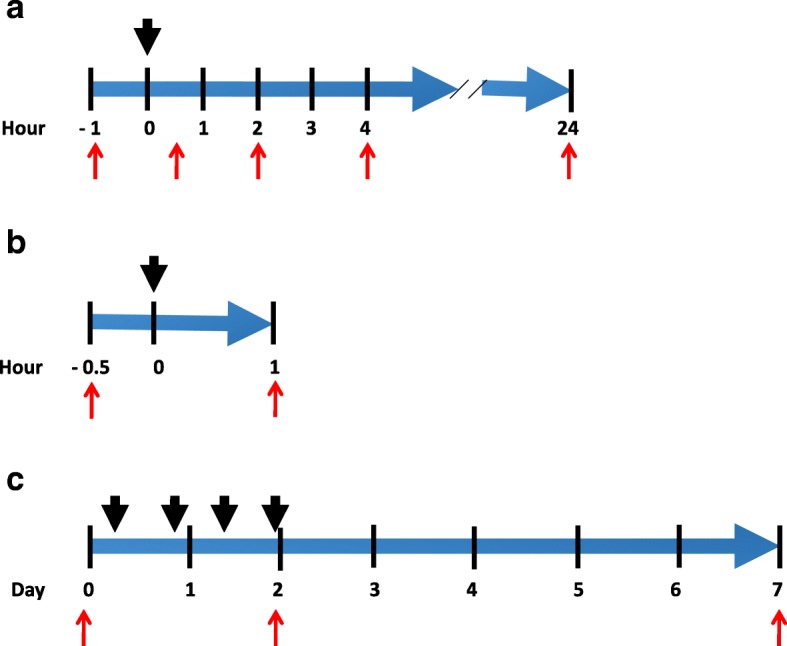
Timeline of investigational studies. (**a**) Study design for healthy subjects at AMC; (**b**) Study design for healthy subjects at FIMR; (**c**) Study design for RA patients at FIMR. Black arrows indicate application of vibrotactile treatment. Red arrows indicate time of blood draws for healthy subjects (**a**, **b**) and clinical assessments for RA patients (**c**)

Human subjects participated at two institutional sites, the Academic Medical Center (AMC), University of Amsterdam, Amsterdam, The Netherlands, and the Feinstein Institute for Medical Research (FIMR), Manhasset, New York, USA. A third cohort of subjects with rheumatoid arthritis were treated at the Feinstein Institute for Medical Research. The study at the AMC was approved by the Institutional Review Board (IRB) of the University of Amsterdam (IRB# MEC 07/095), and the study protocols for healthy subjects (IRB# 11-122B) and RA patients (IRB# 06.02.027) at the FIMR were approved by the Clinical Research Center (CRC) and the Institutional Review Board of Northwell Health, and performed at the CRC of Northwell Health. Healthy subjects at the AMC received vibrotactile stimulation at the cymba concha of the ear (Fig. 1). Blood was collected pre-stimulation and at 30 min, 2 h, 4 h and 24 h post-stimulation from these subjects, and subjected to the whole blood assay (Fig. 2). A randomized cross-over study design was used for the healthy subjects at the FIMR site. Healthy subjects participated in two visits, separated by at least one week. At each visit, the subjects received vibrotactile stimulation either at the cymba concha or at the gastrocnemius muscle (control) using a cross-over design. The subjects were instructed that both the types of treatment were comparable types of nerve stimulations, and not informed about the order of the respective treatment. All treatments with the vibrotactile device on the cymba concha or gastrocnemius were performed between 9:30 a.m. and 11:30 a.m. A washout period of at least one week, but no more than two weeks, was observed between the two stimulations. Blood was collected pre-stimulation and at 1 h post-stimulation during both visits, and subjected to the whole blood assay. RA patients were admitted to the CRC inpatient unit at the Northwell Health for a 48-h period, and received vibrotactile treatment at the cymba concha twice daily (8:00 a.m. and 8:00 p.m.) for two days. Clinical assessments were performed by the same examiner at the time of admission, 48 h and 7 days after vibrotactile treatment.

### Subjects

The study population of healthy subjects at the Academic Medical Center, University of Amsterdam, The Netherlands consisted of 6 participants. The study population at the Feinstein Institute for Medical Research consisted of two groups: healthy subjects (*n* = 19) and RA patients (*n* = 9). Healthy subjects were enrolled to determine whether vibrotactile stimulation inhibits TNF, IL-1β, and IL-6 production in whole blood assay. Informed consent was obtained from all the study participants. Individuals of both genders between the ages of 18 and 60 years were screened. Exclusion criteria were a history of smoking, ear infection (otitis media or otitis externa), arrhythmia, coronary artery disease, chronic inflammatory disease, anemia, malignancy, depression, connective tissue disease (osteoarthritis, vasculitis), neurologic disease, diabetes mellitus, renal disease, malignancy, dementia, psychiatric illness including active psychosis, or any other chronic medical condition. In addition, subjects using cholinergic, anti-cholinergic, or beta-blocking medications were excluded, as were pregnant patients. Study subject characteristics are summarized in Table 1. Nine rheumatoid arthritis patients were recruited from the North American Rheumatoid Arthritis Consortium and by affiliated rheumatology clinic referrals. Exclusion criteria were a history of smoking, immunosuppressive condition (including malignancy and chronic alcoholism), severe dementia, psychiatric illness with active psychosis, current intravenous or other serious illicit drug use, ischemic cardiovascular disease (including myocardial infarction, unstable angina, and bradytachyarrythmias), moderate or severe anemia, pregnancy, and use of anti-cholinergic or beta-blocking medications. Three RA patients had hypertension controlled with medication. No other cardiovascular risk factors were identified in the RA subjects. Patients had an established diagnosis of RA according to American College of Rheumatology criteria. Two patients were excluded from data analysis as they had DAS28 scores at baseline indicating low disease activity.

**Table 1:**
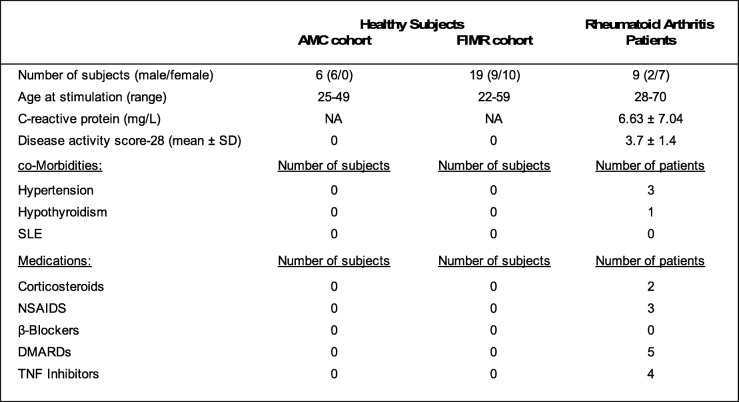
Study participant characteristics

*AMC* Amsterdam Medical Center, *FIMR* Feinstein Instituted for Medical Research CP and Disease activity score-28 are expressed as mean± SD, *NA* Not Available

Subjects participated in three studies at two institutional sites. Six healthy subjects were enrolled at the Academic Medical Center in Amsterdam for a pilot study. Nineteen healthy subjects were enrolled in a controlled cross-over study at the Feinstein Institute for Medical Research. Nine patients with RA were enrolled at the Feinstein Institute for Medical Research

### Vibrotactile device treatment

For the stimulation at the external ear in the healthy subjects and RA patients, the stimulation with the vibrotactile device was delivered at the right cymba concha. For the study of healthy subjects at the AMC, subjects underwent a one-time stimulation of two minutes duration at the right cymba concha. For the study of healthy subjects at FIMR, a cross-over design was utilized in which each subject received a one-time stimulation of two minutes duration at the right cymba concha, and a one-time stimulation of two minutes duration at the right gastrocnemius. RA patients received a total of four stimulations of five minutes duration each using a vibrotactile device (Brookstone). Two stimulations were delivered on the first day, and two stimulations were delivered on the second day. After cleansing the skin with a disposable alcohol prep pad, the device was placed in direct contact with the skin either at the right cymba concha or at the posterior aspect of the leg at the right gastrocnemius muscle (three inches inferiorly from the head of the fibula, and two inches posteriorly), and the treatment was delivered as indicated.

### Disease activity in RA patients

Disease activity in RA patients was measured using DAS28-CRP with four variables, a validated combined index that includes enumeration of tender and swollen joints, visual analog score (VAS), and measurement of high-sensitivity CRP (Fransen et al. 2003). Joint tenderness and swelling were assessed by a single physician to limit variability. The score was calculated as follows: DAS28-CRP = 0.56*sqrt(TJC28) + 0.28*sqrt(SJC28) + 0.36*ln(CRP + 1) + 0.014*GH + 0.96 (https://www.das28.nl/das28/en/). A DAS score of less than 2.6 indicates remission; a score between 2.6 and 3.2 indicates low/minimal disease activity; a score between 3.2 and 5.1 indicates moderate activity; a score of more than 5.1 is considered high/severe disease activity (Fransen and van Riel 2009; Anderson et al. 2012).

### Whole blood assay

Venous blood was drawn into either sodium heparin tubes (AMC) or EDTA tubes (FIMR) (Becton Dickinson, NJ, USA). Blood was aliquoted (0.5 ml) in the assay tubes and stimulated with endotoxin. Endotoxin [lipopolysaccharide (LPS) at FIMR: *Escherichia coli* 0111:B4, Sigma cat. no. L4130 or at AMC: ultrapure, Invivogen, cat. no. tlrl-pelps) was re-suspended to 5 mg/ml, sonicated for 30 min, vortexed, and diluted with RPMI (at AMC) or PBS (at FIMR) to generate a working 1 mg/ml stock. This stock was serially diluted with 1x PBS to final concentrations of 1 ng/ml, in 500 μL blood aliquots. Microfuge tubes aliquoted with blood and endotoxin were incubated on a rocking platform at 37 °C with 5% CO_2_. After a 3-4 h incubation, plasma was collected by centrifugation [5 min, 2000 g (5000 rpm in Microfuge 5415C; Brinkmann, Westbury, NY)] and frozen at − 20 °C for future analysis. All assays were performed in duplicate.

### Cytokine analysis

Interleukin (IL)-6, IL-1β, and TNF levels were determined by using the Cytometric Bead Array (from BD Biosciences) or the V-PLEX proinflammatory panel 1 human kit (Meso Scale Discovery, Gaithersburg, MD, USA), according to manufacturer’s instructions. TNF levels in plasma samples were analyzed by commercially available ELISA kits (R&D Systems, Minneapolis, MN) according to manufacturer’s instructions. High sensitivity CRP serum analysis was performed using a Hitachi 917 automated analyzer (Roche Diagnostics, Indianapolis, IN) at the core laboratory at Northwell Health. The reference value for hsCRP is 0.0–3.0 mg/L.

### Statistical analysis

All statistical analyses were carried out using GraphPad Prism 6 software (GraphPad Software, La Jolla, CA). Data are presented as mean ± SEM where applicable. Repeated measures analysis of variance (RMANOVA) with the Friedman test was used to determine if healthy subjects or RA patients behaved differently over time (pre- vs. post-taVNS). For all analyses, the standard assumptions of Gaussian distribution were tested. The mean changes in TNF release between pre- and post-taVNS or pre- and post-gastrocnemius stimulation in healthy subjects were assessed for significance using a paired Student’s t test. All *p*-values and n values are indicated in figure legends. *P*-values < 0.05 were considered significant.

## Results

### Application of a vibrotactile device at the cymba concha decreases TNF in a pilot study of healthy subjects

**Fig. 3:**
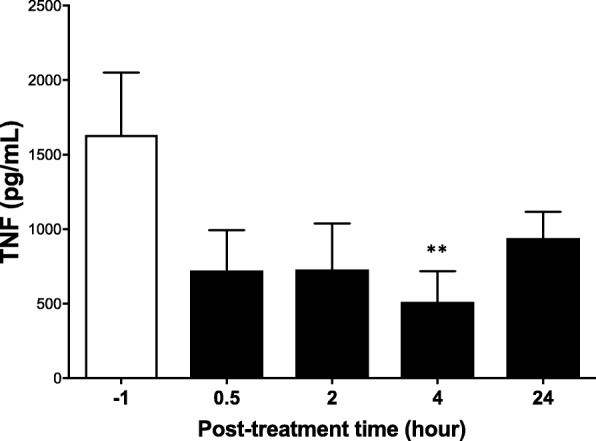
Vibrotactile stimulation at the cymba concha reduces whole-blood LPS-induced TNF production. In a pilot study, six healthy subjects were enrolled at the Academic Medical Center, the Netherlands, and subjected to vibrotactile stimulation at the cymba concha. Blood was obtained before stimulation (− 1) and at different time points after stimulation (0.5, 2, 4 and 24 h). Whole blood was incubated with LPS, and TNF levels in plasma were determined after 4 h in culture. Data are shown as mean ± SEM, **p < 0.01; RMANOVA with Friedman test

To assess the effect of vibrotactile treatment on modulating inflammatory cytokines in healthy subjects, we conducted a pilot study at the Academic Medical Center, University of Amsterdam, Amsterdam, The Netherlands. Vibrotactile treatment was applied to the cymba concha in 6 healthy subjects (6 males, age range 25–49 years, Table 1). No adverse effects after vibrotactile stimulation was reported. TNF production in response to endotoxin challenge was analyzed in the whole blood (Rosas-Ballina et al. 2009) pre- and post- vibrotactile stimulation. We observed that vibrotactile stimulation at the cymba concha in healthy subjects significantly attenuated endotoxin induced TNF by 56, 55, 69% (*p* < 0.01, RMANOVA- Friedman test), and 42% at 30 min, 2 h, 4 h and 24 h post-stimulation respectively (Fig. 3). Interestingly, the suppressive effect persisted up to 24 h post-stimulation (pre-treatment = 1633 ± 417, *n* = 6 vs. 24 h post-treatment = 940 ± 177, *n* = 6).

### Application of a vibrotactile device at the cymba concha, and not the gastrocnemius, attenuates inflammatory responses in healthy subjects

**Fig. 4:**
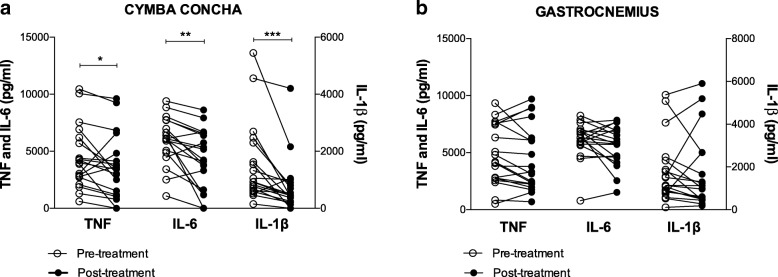
Vibrotactile stimulation at the cymba concha but not at the gastrocnemius, attenuates inflammatory responses in healthy subjects ex vivo. Nineteen healthy subjects enrolled in a cross-over study at the Feinstein Institute for Medical Research received stimulation at either the cymba concha or gastrocnemius on two different days. Blood was collected 30 min prior and 1 h post stimulation. Whole blood was incubated with LPS, and cytokine levels in plasma were determined 4 h after. (**a**) Effect of vibrotactile stimulation at the cymba concha on cytokine responses in healthy subjects. (TNF *p < 0.05, IL-6 ***p < 0.001, IL-1β **p < 0.01, Wilcoxon matched-pairs test) (**b**) Effect of vibrotactile stimulation at the gastrocnemius on cytokine responses in healthy subjects. Open circles represent the pre-treatment and close circles represent post-treatment cytokine levels with lines connecting data points for each individual study participant

Having established the time kinetics for the suppression of inflammatory cytokines in the pilot study of healthy subjects at the Amsterdam site, we next conducted a blinded cross-over study at Northwell Health in Manhasset, NY, USA. In this controlled study, we enrolled 19 healthy subjects (9 males, 10 females; age range 22–59 years of age, Table 1) to determine whether vibrotactile stimulation inhibits TNF, IL-1β, and IL-6 production in whole blood assay. All subjects were scheduled for two visits, separated by at least a week, and received stimulation at either the cymba concha or gastrocnemius. No adverse effects after vibrotactile stimulation at either anatomical location were reported, and the stimulation was well-tolerated. Peripheral blood was collected 30 min before and 1 h after the stimulation, and subjected to endotoxin stimulation in a whole blood assay. As shown in Fig. 4a, vibrotactile stimulation at the cymba concha significantly reduced TNF, IL-1β, and IL-6 levels compared to pre-stimulation baseline (TNF *p* < 0.05, IL-1β *p* < 0.001, IL-6 *p* < 0.01, Wilcoxon matched-pairs test), whereas stimulation at the gastrocnemius did not attenuate inflammatory responses. Vibrotactile stimulation at the cymba concha inhibited TNF by 20% (pre-stimulation = 4541 ± 624 pg/ml vs. post-treatment = 3625 ± 645 pg/ml), IL-6 by 27% (pre-stimulation = 5979 ± 480 pg/ml vs. post-treatment = 4342 ± 597 pg/ml), and IL-1β by 50% (pre-treatment = 1527 ± 328 pg/ml vs. post-treatment = 765 ± 222 pg/ml) as compared to the baseline levels before treatment. The attenuation of cytokine levels following vibrotactile treatment of the cymba concha cannot be attributed to a placebo effect, as vibrotactile treatment at the gastrocnemius for one minute did not change endotoxin-induced TNF (pre-treatment = 4796 ± 607 pg/ml vs. post-treatment = 4477 ± 653 pg/ml), IL-6 (pre-treatment = 6137 ± 369 pg/ml vs. post-treatment = 5577 ± 401 pg/ml) and IL-1β (pre-treatment = 1787 ± 344 pg/ml vs. post-treatment = 1667 ± 398 pg/ml) levels in whole blood (Fig. 4b). To our knowledge, this is the first report that application of a vibrotactile device at the cymba concha inhibits endotoxin-induced whole-blood TNF, IL-1β, and IL-6 in humans.

### Application of a vibrotactile device at the cymba concha decreases disease activity scores in patients with rheumatoid arthritis

**Fig. 5:**
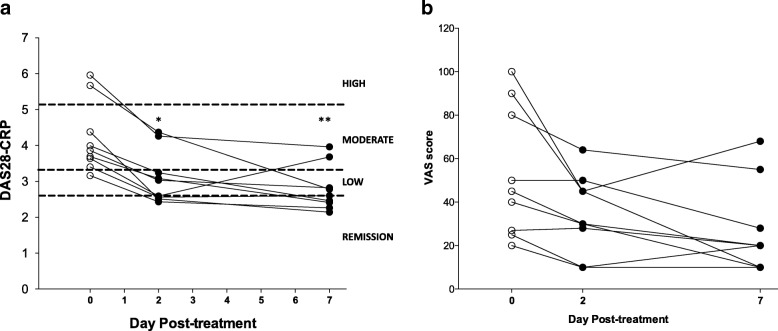
The effect of vibrotactile treatment at the cymba concha in patients with rheumatoid arthritis. (**a**) The DAS28-CRP scores, (**b**) The visual analog scores in nine RA patients enrolled in the prospective interventional study. DAS28-CRP scores indicate a composite score of enumeration of swollen and tender joints, measurement of high-sensitivity CRP in serum, and scores on the visual analog scale, a validated patient-reported assessment of functional status and well-being. These data were obtained at day 0 (pre-treatment), day 2 and day 7 (post-treatment) following vibrotactile treatment at the cymba concha. Lines connect data points for the DAS28-CRP score of each individual study participant at these timepoints. Hashed lines indicate the cut-off points for categories of disease severity. A DAS score of less than 2.6 indicates remission; a score between 2.6 and 3.2 indicates low/minimal disease activity; a score between 3.2 and 5.1 indicates moderate activity; a score of more than 5.1 is considered high/severe disease activity (Fransen and van Riel 2009; Anderson et al. 2012). The significance of the change by RMANOVA using Friedman test between visits is shown: *p < 0.05 vs. day 0; **p < 0.01 vs. day 0)

Next we investigated whether vibrotactile treatment confers clinical benefit to rheumatoid arthritis patients. Nine RA patients with active disease (DAS-28 score 4.19 ± 0.33) were enrolled in the study and received vibrotactile treatment twice daily for two days. Disease activity was reassessed 2 days and 7 days post-vibrotactile treatment. As shown in Fig. 5a, vibrotactile treatment at the cymba concha significantly decreased DAS-28 scores in RA patients at two days post-treatment (DAS28 score pre-treatment = 4.19 ± 0.33 [3.16–5.96] vs. post-treatment = 3.12 ± 0.25 [2.43–4.37], *p* < 0.05, RMANOVA, Friedman test). Disease activity remained significantly reduced 7 days following vibrotactile treatment (DAS28 score 7 days post-stimulation = 2.79 ± 0.21 [2.14–3.96]; *p* < 0.01, RMANOVA, Friedman test).

At the time of the study, nine RA patients had active disease (moderate to severe, DAS28 score > =3.2) based on the criteria by American College of Rheumatology (Anderson et al. 2012); two additional patients had inactive disease (DAS28 score < 2.6) and underwent the study, but these data were not included as part of the analysis. Disease activity was significantly attenuated in RA patients with active disease (24–51% reduction in DAS28 scores over 7 days) after vibrotactile treatment at the cymba concha. 85% of the patients with moderate disease (5 out of 7 patients DAS28 scores 3.16–4.38) reached inactive disease stage by 7 days (DAS28 scores 2.14–2.41); whereas in two of the study subjects with severe disease (100%, 2 out of 2 subjects), vibrotactile treatment at the cymba concha reduced disease activity by 34–51% (DAS28 score 5.67 and 5.96 reduced to 2.78 and 3.96 respectively). Following taVNS, circulating CRP levels were significantly reduced after 2 days (pre-treatment = 6.66 ± 2.5 [0.2–21] vs. post-treatment = 4.71 ± 1.71 [0.3–14], *p* < 0.05, RMANOVA, Friedman test) but returned back to the baseline after 7 days (6.97 ± 2.56 [0.7–16.2]).

### Application of a vibrotactile device at the cymba concha decreases global health VAS in patients with rheumatoid arthritis

Self-reported well-being and pain are routinely used in the assessment of RA (Amaya-Amaya et al. 2012). Vibrotactile treatment of the cymba concha reduced visual analogue scale (VAS) scores, a patient derived measure of global health assessment when assessed 2-days post-treatment (VAS score pre-treatment = 53.0 ± 9.9 vs. post-treatment = 34.7 ± 6.0; *p* < 0.05, RMANOVA, Fig. 5b); this effect persisted throughout the 7 day study period (VAS score 26.8 ± 6.95; p < 0.05, RMANOVA, Fig. 5b). Together, these results indicate application of a vibrotactile device to the cymba concha of the external ear may confer therapeutic benefit to RA patients.

## Discussion

Our results demonstrate that vibrotactile stimulation at the cymba concha attenuates inflammatory responses in the settings of both health and disease, and further suggest that the auricular branch of the vagus nerve is a functional component of the inflammatory reflex. In healthy subjects, vibrotactile stimulation at the cymba concha attenuated endotoxin induced TNF responses in whole blood assay for up to 24 h. Using a cross-over study, we found that vibrotactile stimulation at the cymba concha but not at the gastrocnemius reduces TNF, IL-6, and IL-1β responses ex vivo following endotoxin challenge in healthy patients, and attenuates disease severity in RA patients with moderate or severe disease. Reduction of systemic inflammation in patients may be achieved by delivering electrical impulses to the vagus nerve at the cervical level (Koopman et al. 2016; Bonaz et al. 2016). Moreover, in healthy subjects, transcutaneous cervical vagus nerve stimulation decreases cytokines and chemokines in whole blood cultures (Lerman et al. 2016). These signals travel distally to the spleen, where they terminate on acetylcholine-synthesizing T cells, which in turn inhibit the inflammatory responses of splenic macrophages (Rosas-Ballina et al. 2011). Previous work has anatomically demonstrated projections of the auricular branch of the vagus. Activation of the NTS has been demonstrated following vibrotactile treatment of the cavum concha in rats (Ay et al. 2015), and afferent fibers of the vagus are known to terminate primarily in the nucleus tractus solitarius (Goehler et al. 2000). The modulation of immunological endpoints both in endotoxin assays performed on blood from healthy subjects and attenuation of disease severity in RA patients strongly suggests that the vibrotactile stimulus at the cymba concha in this study induces neural signals which converge on the efferent neural signaling pathway of the inflammatory reflex. While the origin of descending motor fibers in the vagus that ultimately target the spleen was beyond the scope of the present study, it is nonetheless a subject of interest for future study.

The stimulation modality, anatomical location, and treatment duration in the present study are different in important ways from these previous studies. The auricular branch of the vagus nerve supplies several regions of the auricle with sensory fibers. The cymba concha region has been shown by cadaveric studies to receive all of its sensory innervation from the auricular branch of the vagus nerve, with no additional innervation from other cutaneous nerves (Peuker and Filler 2002). Integration of anatomical studies, functional studies, and the modulation of clinical endpoints by different approaches to vibrotactile treatment will be important to define both the neuroanatomy and biology of the auricular branch of the vagus nerve as well as its role as a therapeutic target.

While this study was not designed to assess the dose-dependence of vibrotactile treatment, it is interesting to note that the disease attenuation observed in RA patients – as indicated by both DAS28 scores and VAS scores – persisted for up to 7 days in the majority of patients. The RA patients in this study received a total of 4 separate stimulations, each lasting 2 min, delivered on two sequential days, totaling 8 min of vibrotactile treatment in sum. That the reduction of TNF, IL-6 and IL-1 β observed in ex vivo assays following endotoxin challenge in healthy subjects were likewise achieved with minimal stimulation duration. These results demonstrate that in the absence of systemic inflammation, the motor arm of the inflammatory reflex may still be targeted in healthy subjects to reduce the levels of inflammatory cytokines below their physiological set points.

The primary objective of this study was to determine whether activating the auricular branch of the vagus nerve using vibrotactile device inhibits cytokines in humans and improves disease severity in RA. It is reasonable to consider whether placebo mechanisms contribute to these findings as study subjects are aware when the device is delivering the stimulation. It is plausible that placebo mechanisms may contribute to attenuating disease scores in RA. However, lack of any cytokine suppressing effects following vibrotactile stimulation at the gastrocnemius in healthy subjects argues against the placebo effect. A related and open question regarding the nature of vagus nerve activation is the identity of the fibers that carry the signals which culminate in anti-inflammatory effects. Trains of electrical taVNS have been shown to modulate heart rate in a parameter-dependent fashion (Badran et al. 2018). It follows therefore, that similar parametric determinants of vagus nerve signaling may differentially regulate inflammatory cytokines by engaging different types of fibers, a concept which we recently demonstrated in afferent cytokine-mediated signaling of the vagus nerve (Zanos et al. 2018). A limitation of our study is the inability to control for the sound of the vibrotactile device during simulation. These sound waves are produced in close proximity to the external auditory meatus, and could simultaneously engage other neural pathways. Whether sound waves themselves can activate neural signals to control immune responses is an additional interesting subject of study. Taken together, the data in this study support the mechanistic and therapeutic framework for the use of bioelectronic devices to target neural circuits previously mapped to control inflammatory responses. Vibrotactile taVNS warrants further study for the treatment of RA and other disorders of systemic inflammation, and may be of particular relevance in patients for whom implantable vagus nerve stimulators are not tolerated or are otherwise contraindicated or unavailable.

## Conclusion

Together, these studies establish that vibrotactile stimulation at the cymba concha modulates TNF, IL-6 and IL-1β production and reduces inflammation in humans. These findings also demonstrate that transcutaneous vagus nerve stimulation reduces the disease severity in RA patients. This pilot study supports the future development of novel non-invasive bioelectronic treatment modalities for diseases currently treated with drugs. Clinical trials in RA are warranted to address the clinical efficacy, as our findings suggest that it is possible to use the vibrotactile taVNS in the experimental therapy of RA and possibly other cytokine-mediated auto-inflammatory disorders.

## Abbreviations

AMC: Academic Medical Center
CRC: Clinical Research Center
CREB: cAMP response element-binding protein
DAS: Disease activity score
EDTA: Ethylenediaminetetraacetic acid
ELISA: Enzyme-linked immunosorbent assay
FIMR: Feinstein Institute for Medical Research
hsCRP: High-sensitivity c-reactive protein
IL-1β: Interleukin-1 beta
IL-6: Interleukin-6
LPS: Lipopolysaccharide
NFκB: Nuclear factor kappa-light-chain-enhancer of activated B cells
RA: Rheumatoid arthritis
RMANOVA: Repeated measures analysis of variance
taVNS: Transcutaneous auricular vagus nerve stimulation
TNF: Tumor necrosis factor
VAS: Visual analog scale
VDC: Volts direct current
α7nAChR: α7 nicotinic acetylcholine receptor

## Authors’ information

There is no additional author information to be included.
